# 

**Published:** 1821-03-01

**Authors:** 


### Contents

OF

### No. 4. FOR MARCH, 1821.

ART. I.

A Treatise on Verminous Diseases, preceded by the Natural History of Intestinal Worms, &c. &c. By Professor BRERA - - - - - - - - - - - - - - - - - - 556

II.

An Essay on Syphilis. By GEORGE BALLINGALL, M. D. - 581

III.

A Treatise on the Plague, designed to prove it contagious, &c. By Sir ARTHUR BROOKE FAULKNER, M. D. - - - 586

IV.
The Principles of Midwifery, including the Diseases of Women and Children. By JOHN BURNS, R. P. &c. *New Edit.* 595

V.

1. Dr. SCUDAMORE's Chemical and Medical Report on Mineral Waters. - - - - - - - - - - - - - - - - - 605

2. Dr. MACKENZIE on Mineral Waters *Second Edition* . 605

VI:

MEDICAL TRANSACTIONS OF THE COLLEGE OF PHYSICIANS IN LONDON. Vol. VI. (Concluded.) - - - - - - - 622

Art. IX. Dr. PHILIP on the Calculous Diathesis. - - - - 623

X. Dr. GOOCH on Spontaneous Evolution of the Fœtus 627

XI. Dr. LATHAM on Venesection - - - - - - - 629

XII. Dr. GOOCH on Puerperal Insanity - - - - - 631

XIII. Dr. YEATS on the Duodenum - - - - - 639

XIV. Dr. DOUGLAS on Hour-Glass contraction of the Uterus - - - - - - - - - - - - - - - - 647

XV. Sir HENRY HALFORD on Cautious Prognosis, in certain Diseases - - - - - - - - - - - - - 650

XVI. Mr. STANLEY on Superfœtation - - - - - 654

VII.
Dr. ELLIOTSON on Hydrocyanic Acid, in Affections of the Stomach, Lungs, &c. &c. - - - - - - - - - - - - 655

VIII.
Dr. M'CABE on the Cheltenham Waters, and the Diseases in which they are recommended - - - - - - - - - - 665

IX.
A Practical Treatise on the Diseases of the Eye. By JOHN VETCH, M. D. F. R. S. E. - - - - - - - - - - - 668

X.

Mr. WHITER's Dissertation on the Disorder of Death, or that State of the Frame termed—"Suspended Animation." - 687

XI.

Dr. FORBES on the Climate of Penzance and District of the Land's End - - - - - - - - - - - - - 695

XII.

Dr. DAVIES on Mercury - - - - - - - - - - - - - 700

XIII.

Dr. GRANVILLE on the Internal Use of Prussic Acid. *2d Edit.* 702

XIV.

VACCINATION.

1. Sir GILBERT BLANE on Vaccination - - - - - - - }
2. Report to the Secretary of State on Vaccination - - - } 718
3. Dr. JENNER's Circular on Vaccination - - - - - - - }

XV.

Dr. JOHN COOKE on the various Species of Palsy - - - 725

XVI.

Mr. ARNOTT on Obstructions in the Urethra, Rectum, &c. (with a plate) - - - - - - - - - - - - - - 743

XVII.

SUPPLEMENTAL REVIEW, AND QUARTERLY PERISCOPE OF PRACTICAL MEDICINE, SURGERY, &c. - - - - - - 757

1. Mr. PATTISON on Internal Aneurisms - - - - - - - 757
2. Dr. DICKSON on Erysipelas - - - - - - - - - - - 761
3. Dr. SHERRIL on Disease of the Heart - - - - - - - 762
4. M. VAIDY on Tic Douloureux - - - - - - - - - - 764
5. Dr. ABERCROMBIE on Pseudo-Consumptive Diseases - 767
6. Dr. WILLIAMS on Vinum Seminis Colchici - - - - 769
7. Dr. ROBERTSON on Hooping Cough - - - - - - - 770
8. Dr. ROBBINS on Disease of the Heart - - - - - - 771
9. Ruptured Tendo Achillis - - - - - - - - - - - 773
10. Serious Affection of the Stomach from drinking Cold Water 773
11. Ligature of the common Iliac Artery - - - - - - - 774

XVIII.

MISCELLANIES.

Remarks on Medical Criticism - - - - - - - - - - - 778
Death of Dr. BAYNTON - - - - - - - - - - - - - - *ib.*
Dr. MAGENDIE's Experiments on Hydrophobia - - - - 779
New Journal of Physiology - - - - 780
Dr. JENNER's Circular Letter on Vaccination - - - - - - 780
Dispensary for Cutaneous Diseases - - - - - - - - 784
Bibliographical Record - - - - - - - - - - - - - - 784
General List of Subscribers - - - - - - - - - - - - 793
Dr. GRANVILLE's Reply to the Journal of Science - - - 803
Intelligence, &c. - - - - - - - - - - - - - - - - - 818

